# Revolutionizing breast cancer immunotherapy by integrating AI and nanotechnology approaches: review of current applications and future directions

**DOI:** 10.1186/s42234-025-00173-w

**Published:** 2025-05-30

**Authors:** Houda Bendani, Nasma Boumajdi, Lahcen Belyamani, Azeddine Ibrahimi

**Affiliations:** 1 Laboratory of Biotechnology Lab (MedBiotech), Bioinova Research Center, Rabat Medical and Pharmacy School, Mohammed V University in Rabat, Rabat, Morocco; 2 Mohammed VI Center for Research and Innovation (CM6), Rabat, Morocco; 3 Mohammed VI University of Sciences and Health (UM6SS), Casablanca, Morocco; 4 Emergency Department, Military Hospital Mohammed V, Rabat Medical and Pharmacy School, Mohammed V University, Rabat, Morocco

**Keywords:** Breast cancer, Immunotherapy, Artificial intelligence, Personalized medicine, Multi-omics, Decision-making, Nanomedicine, Biomarkers

## Abstract

Breast cancer (BC) is still the most diagnosed cancer for females with an increased focus on immunotherapy as a promising precise treatment. Selecting appropriate patients and monitoring patient treatments are crucial to ensure higher response rates with low adverse events. Various biomarkers were proposed to predict immunotherapy response, including tumor mutation burden, immune cell, and tumor microenvironment expression. However, traditional methods for evaluating immunotherapy are invasive and inaccurate, and their assessments could be biased due to the variability in quantification techniques. Artificial intelligence (AI) has emerged as a powerful technology that addresses these challenges, handling heterogeneous data to identify complex patterns and offering accurate and non-invasive solutions. In this paper, we review emerging AI-based models for immunotherapy prediction in BC using diverse biomarkers. We first discussed the application of AI models for each biomarker, highlighting both direct prediction of immunotherapy response and prognosis, as well as indirect approaches via the identification of immune subtypes or specific predictive biomarkers. Then, we investigated the integration of all biomarkers in multi-modal AI approaches for a precise and personalized prediction of immunotherapy response. We have also addressed the implication of integrating AI in the healthcare ecosystem with other new technologies, including nanodevices, and wearable technologies. We further elucidated the role of AI and healthcare providers with this convergence of personalized medicine and demonstrated its role in enhancing population health management and supporting personalized patient care.

## Background

Breast cancer (BC) is the most diagnosed cancer occurring among women, with 2.29 million new cases and 666,103 deaths in 2022, based on the latest Global Cancer Observatory Statistics. With 11.6% of all cancers, new cases in BC are estimated to reach 2.42 million by 2025 (Kim et al. 2025). BC is identified based on the immunohistochemical measures of estrogen receptor (ER), antigen Ki-67 (Ki-67), human epidermal growth factor receptor 2 (HER2), and progesterone receptor (PR) (Cuzick et al. 2011). Luminal A (ER/PR +, HER2 −, Ki67 + < 20%) is the most frequent subtype of BC, characterized by a lower proliferation rate and a better prognosis with high overall and disease-free survival rates. In contrast to luminal A, luminal B (ER/PR + < 20%, HER2 −, Ki67 + ≥ 20%) is an intermediate/high histologic grade representing 15–20% of all BCs. The elevated Ki-67 and other molecular mechanisms contribute to this subtype's aggressiveness and poorer prognosis. Triple-negative BC (TNBC) is the most challenging subtype, accounting for less than 15% of BC subtypes. TNBC is more aggressive with high recurrence and mortality rates (Barzaman et al. 2020; Abubakar et al. 2019; Goldhirsch et al. 2013).

Due to the heterogeneity of BC, treatment options vary according to the subtype, stage, grade, and some specific molecular markers. Therefore, efforts have been invested in developing new treatment strategies that guarantee better survival. Surgery is part of the traditional treatments, while chemotherapy and immunotherapy are considered personalized treatments. Immunotherapy, especially immune checkpoint inhibitors (ICIs), has recently shown promising results in cancer treatment. By enhancing the body’s natural immune system, it targets cancer cells and blocks checkpoint proteins. Programmed cell death receptor 1 (PD-1) and programmed cell death 1 ligand 1 (PD-L1) are the widely approved inhibitors along with the cytotoxic T lymphocyte-associated antigen (CTLA-4) (Shiravand et al. 2022; Pardoll 2012). Other studies investigate novel inhibitors such as lymphocyte activation gene-3 (LAG-3), VISTA, and B7-H3 (Rivoltini et al. 2024; Dirix and Triebel 2019; Curigliano et al. 2021; Lee et al. 2017).

Although ICIs have breakthroughs and promising results in cancer treatment, ongoing research is still needed to overcome its limitations such as immune-related adverse effects (irAEs), resistance, tolerance of therapy, upregulation of inhibitory pathways, and inconsistency in treatment response. An estimated rate of 43.6% of cancer patients in the USA were eligible for ICI and only 12.5% responded to it (Haslam et al. 2020). This variability in response is mainly due to the patient's clinical and tumor heterogeneity. TNBC is known for its immunogenic and heterogenic tumor microenvironment, which regulates the proliferation and metastasis of cancerous cells. The expression of multiple components of the TME has a prognostic role, including tumor-infiltrating lymphocytes (TILs), CD8 + T cells, activated NK cells, and the CD8 +/Treg ratio (Dieci et al. 2021; Tavares et al. 2021). Furthermore, the high cost of ICI drugs/agents, the low response rate to ICI, and severe irAEs highlight the need for reliable biomarkers to stratify patients before receiving ICIs as well as facilitate the identification of optimal alternate treatment protocols for non-responsive patients. PD-L1 expression, such as combined positive score or circulating tumor cells, is the primary and common method to identify patients for ICI (Zhou et al. 2023; Gruosso et al. 2019). However, different clinical trials have been approved to assess PD-L1 levels which can impact patient selection (Qi et al. 2022).

Given those challenges, suitable biomarkers represent a powerful tool for identifying patients eligible for ICIs. PD-L1 expression, tumor mutation burden (TMB), TILs, and microsatellite instability (MSI) or mismatch repair deficiency are some of the biomarkers used to predict response to immunotherapy in BC (Qi et al. 2022; Cui et al. 2023; Presti et al. 2022; Carlino et al. 2022).

However, these conventional biomarkers offer limited predictive power due to unstandardized quantification, the complexity of the tumor immune microenvironment, and its variability. Besides, these biomarkers are often manually assessed and vary depending on the tumor regions, affecting their overall predictive potential.

To address these limitations and further unravel the complexity of cancer immunotherapy, AI has emerged as a powerful tool offering novel strategies to improve biomarker discovery and predict immunotherapy response. AI techniques can compute and integrate high-dimensional data such as genomics, hematology, and transcriptomics to generate immune signatures or discover novel immune subtypes associated with immunotherapy response. Furthermore, as immunotherapy biomarkers like TMB or TILs fail at predicting response across BC subtypes, incorporating AI approaches as multimodal allows the integration of different data types (genomic mutations, clinical data, and imagery) to stratify patients with higher accuracy. Another key advantage of the AI approach is the possibility to model temporal data and longitudinal data, offering a better prediction of resistance mechanisms and simulating their pathways. In the present review, we aim to explore the recent advancements in artificial intelligence (AI) applications in BC immunotherapy, highlighting research across various omics fields.

We conducted a structured literature search across several databases, such as PubMed, Scopus, WOS, and Google Scholar. The search included relevant research articles, conference papers, and reviews published between January 2020 and February 2025. We considered the following inclusion criteria: (1) Any type of immunotherapy or combined with other treatments; (2) Neoadjuvant immunotherapy; (3) Pan-cancer studies that included a breast cancer dataset representing at least 10% of the total samples; and (4) Articles written in English. We used combinations of the relevant keywords and medical subject headings (MeSH) terms for artificial intelligence, immunotherapy, breast cancer, nanotechnology, and computational modeling.

Selected articles were categorized based on their approach into single-omics studies, analyzing single biological data types, and multi-omics studies integrating two or more omics types. The present review is structured as follows: Firstly, we presented AI-based applications in single-omics analyses, including transcriptomic, microbiomic, genomic, epigenomic, proteomic, radiomic, and histopathological data. Multi-omics studies followed, where we discussed how integrative approaches leverage the prediction of immunotherapy response. Subsequently, we explored computational modeling strategies and the application of nanotherapy in BC immunotherapy prediction. Finally, we discussed the limitations and future directions of AI and nanotechnologies.

Table 1 compiles all the referenced studies mentioned in this review, categorized according to their respective omics fields.

**Table 1:** Summary of AI methods in breast cancer immunotherapy prediction

Omic	Biomarker	Task	Data source	Tumor	Model/Algorithm	Year	Reference
**Transcriptomic**	Expression of M2-like tumour-associated macrophages	Immunotherapy response	TCGA	TNBC	Simple neural network-based deep learning	2020	Bao et al. 2020)
	Expression of antigen processing and presentation machinery	Prognostic	TCGA	BC	LASSO logistic regression and RF	2023	Müller et al. 2023)
	Expression of costimulatory molecule genes	TIME status	TCGA	TNBC	LASSO and SVM-RFE	2024	Zhang et al. 2024)
	Expression of genes	Immune-related cells	Institutional and public Dataset	BC and melanoma	Gaussian mixture modeling, Kullback–Leibler (KL) divergence, and mutual nearest-neighbors criteria	2023	Zou et al. 2023)
	Expression of redox-related gene	Prognostic	Institutional and public dataset	BC	RF, LASSO, GBM, Survival-SVM, superpc, Ridge Regression, plsrcox, coxboost, Stepwise Cox regression, and Enet	2024	Wang et al. 2024)
	Expression of genes	pCR	Institutional and public dataset	BC	LASSO, SVM-RFE, and xgboost	2024	Lu et al. 2024)
	Expression of DPP4-related genes	Prognosis	Institutional and public dataset	TNBC	Xgboost, RF, adaboost, KNN, ANN, and LR	2023	Kang et al. 2023)
**Proteomic**	IHC and H&E TMA	PD-L1 expression	Public dataset	Breast cancer	CNN model with residual connections	2022	Shamai et al. 2022)
	IHC images	PD-L1 Status	Institutional dataset	Pan-cancer	Weakly supervised deep learning with ResNet-50 and attention-based MIL	2024	Ligero et al. 2024)
	Expression of signature proteins	Immunotherapy response	Institutional dataset	TNBC	RF	2024	Li et al. 2024)
**Pathology**	H&E WSI	Prognostic	Institutional and TCGA dataset	TNBC	Dual CNN	2022	Zhao et al. 2022)
	WSI	TIL	Clinical studies and TCGA datasets	BC	CNN architectures with segmentation and attention-based classifier	2024	Perera et al. 2024)
	WSI	Immunotherapy-related gene	TCGA	BC	MIL architecture with resnet-18	2025	Zhang et al. 2025)
	H&E TMA	pCR	Clinical studies	TNBC	Regularized logistic regression	2023	Wang et al. 2023a)
	H&E WSI	lncRNA–metabolism class prediction	Institutional and TCGA dataset	BC	Resnet50 for feature extraction and gated attention	2024	Yu et al. 2024)
	H&E WSI	TMB	TCGA	TNBC	LR, KNN, RF, and DT	2025	Bendani et al. 2025)
	H&E WSI	TIL and ki-67	Public dataset	BC	U-net-like backbone with CNN and residual dilated inception module	2021	Negahbani et al. 2021)
**Radiomic**	DEC-MRI	pCR	Institutional dataset	TNBC	Lasso regression	2023	Ramtohul et al. 2024)
	DCE-MRI	TME subtypes	TCGA	BC	RF	2024	Han et al. 2024)
	CE-CT	Immunotherapy response	Clinical studies	BC	Multivariable logistic regression	2023	Zhao et al. 2023)
	MRI	TIL	Institutional dataset	BC	LR, RF, MLP, SVM, LDA, and GB	2022	Huang and Lin 2022)
	PET/CT	pCR	Institutional dataset	TNBC	LR	2023	Seban et al. 2023)
**Epigenetic**	Methylation level of CpG sites	Prognostic	TCGA	BC	Cox and LASSO regression	2021	Zhang et al. 2021)
	Expression of senescence-relevant lncRNAs	Prognostic	TCGA	BC	Cox and LASSO regression	2023	Yu et al. 2023b)
	Expression of nine hub lncRNAs	CD8 T-cell levels	TCGA	BC	DT, GBM, GLM, ANN, RF, SVM	2022	Chen et al. 2022b)
	Expression of Mitochondrial DNA methylation	Prognostic	TCGA	BC	Cox and LASSO regression	2023	Ma et al. 2023)
**Microbiology**	Abundances of microbe	Prognostic	TCGA	BC	Cox and LASSO regression	2021	Mao et al. 2022)
	Genus-level intratumor microbiome	Prognostic	TCGA	BC	Cox and LASSO regression	2024	Li et al. 2024)
**Omic**	256 multi-modal biomarker signatures from (gene expression, cellular morphometric biomarkers, and abundance of microbe)	Prognostic	TCGA	BC	Multivariate Cox regression	2022	Mao et al. 2022)
	Radiological features and gene expression	Axillary lymph node metastasis	TCGA	BC	Multivariate LR analysis	2024	Lai et al. 2024)
	Pathology images, lncRNA data, immune cell scores, and clinical features	Prognostic	Institutional and TCGA dataset	BC	CNN architecture with attention-module, concatenation fusion, and a conclusive regressor	2024	Yu et al. 2024)
	Mammogram, MRI, radiological, histopathological, personal, and clinical data	Pcr	Institutional and public Dataset	BC	Resnet18 architecture for feature extraction, cross-modal knowledge mining, and ensemble model for final prediction	2024	Gao et al. 2024)
**Genomic**	RNA-seq data	Somatic copy number aberrations	TCGA	Pan-cancer	Sequence models and graph neural networks	2024	2024)
	Whole-exome sequencing and RNA sequencing	Neoantigen immunogenicity	Institutional dataset	Pan-cancer	Ensemble model (LR and xgboost)	2023	Jin et al. 2021)
	Genomic instability-related lncRNA signature	Prognostic	TCGA	BC	Multivariate Cox regression	2022	Jiao et al. 2022)

*TCGA* The cancer genome atlas, *TNBC* Triple-negative breast cancer, *BC* Breast cancer, *LASSO* Least absolute shrinkage and selection operator, *RF* Random forest, *SVM-RFE* Support vector machine-recursive feature elimination, *GBM* Gradient boosting machines, *SuperPC* Supervised principal component, plsRcox partial least squares cox regression,*Enet* Elastic net, *xgBoost* extreme gradient boosting, *AdaBoost* Adaptive boosting, *KNN* K-nearest neighbor, *ANN* Artificial neural networks, *LR* Logistic regression, *DT* Decision tree, *MLP* Multilayer perceptron, *LDA* Linear discriminant analysis, *GB* Gradient boosting, *GLM* Generalized linear models, *CNN* Convolutional neural network, *MIL* Multiple instance learning, *xgBoost* extreme gradient boosting, *IHC* Immunohistochemistry, *H&E* Hematoxylin and eosin, *TMA* Tissue microarray, *pCR* pathological complete response, *DPP4 dipeptidyl* peptidase 4, *TIL* Tumor-infiltrating lymphocytes, *TME* Tumor microenvironment, *DCE-MRI* Dynamic contrast-enhanced MRI, *CT* Computed tomography, *PET* Positron emission tomography, *WSI* Whole slide imaging, *TMB* Tumor mutation burden, *lncRNA* long non-coding RNA

### AI-based transcriptomic analysis

AI models have been employed to predict immunotherapy response by leveraging different transcriptomic biomarkers such as immune-related gene signatures, tumor microenvironment (TME) profiles, and subtype-specific gene expression. Transcriptomic data obtained from bulk, single-cell RNA sequencing, or spatial transcriptomics enables AI models to identify predictive genes and understand the cellular dynamics that regulate response or resistance to immunotherapy treatments.

Studies have integrated AI models with transcriptomic data to complete different tasks, from signature prediction to predictive modeling of immunotherapy outcomes. A detailed summary of these studies is presented in Table 1. Some studies have focused on immune infiltration and subtype classification. They used unsupervised clustering and kmers to stratify BC samples into immune subtypes, identify differentially expressed genes (DEGs), and employ models such as random forest (RF) and multilayer perceptron (MLP) to create the predictive model. Other studies developed tumor microenvironment (TME)-associated gene signatures to predict immune checkpoint therapy response by capturing immune cells, tumor cells, stromal cells, and the extracellular matrix. Researchers widely use LASSO, Cox regression, and random survival forests (RSF) for feature selection and prognosis model construction. Gene signatures linked to pathological complete response (pCR) and residual disease (RD) status were also employed to predict ICI responses in BC patients. Another major focus has been on metabolism-related gene signatures, particularly those linked to redox balance, anoikis resistance, glutamine metabolism, palmitoylation, and lactate-hypoxia interactions. Multiple models were applied and compared to create the predictive scores, followed by TIDE or ESTIMATE analysis and correlation with drug sensitivity for validation.

A noticeable remark in these studies is the gene signature formula used, which varies depending on the prediction task. While the majority of studies adopted linear models (LASSO, SVM, and Elastic Net) to compute the signature score using the expression of the genes with their respective coefficients, other studies applied PCA on the genes to construct continuous scores. For instance, Gou et al. constructed a tumor microenvironment-related gene score by summing PC1 and PC2 values derived from PCA on the TME-related genes. This approach allowed the authors to capture multiple dimensions of tumor microenvironment activity, including immune and stromal signals, thereby producing a more comprehensive and biologically informed score to stratify patients (Gou et al. 2022). A different approach was adopted by Lu et al., where they constructed a principal component-based gene score to distinguish patients who can benefit from ICI by identifying the PC1 of the pCR-associated genes (up in responders) and subtracting the PC1 of the RD-associated genes (up in non-responders). By computing both types of expression genes independently, they provided a precise scoring method to better represent the relationship between immune activation and resistance mechanisms and stratify patients who are likely to benefit from immunotherapy (Lu et al. 2024).

​Regarding the model architectures, LASSO, Cox regression, and RF are widely used in clinical gene signatures and for survival prediction, mostly due to their transparency, simplicity, and suitability for high-dimensional data. Model performances range around 0.70 (Zhang et al. 2024; Zhu et al. 2024a; Shen et al. 2023). While these algorithms offer a strong ranking method and a large validation technique, such as cross-validation and C-index, something they lack is non-linear effects, thereby not being able to model complex biological relationships necessitating heavy validation techniques. In contrast to these traditional models, several risk scores were constructed using more advanced or hybrid approaches. AIARS (Wang et al. 2024) and AIDAS (Guo et al. 2024) were computed following an extensive workflow composed of a combination of multiple machine learning algorithms like Random Survival Forest (RSF), LASSO, Ridge, and CoxBoost compared using the mean C-index across multiple cohorts. This demonstrated the utility of ensemble and comparative ML designs in the selection of representative genes while maintaining good generalizability. scCURE, on the other hand, combined Gaussian mixture models and mutual nearest-neighbor criteria to analyze changed and unchanged immune-related cells during immunotherapy in BC patients (Zou et al. 2023). A neural network was constructed to predict immunotherapy based on the expression of signature genes, achieving a 100% area under the curve (AUC) when evaluated in an independent validation set (Bao et al. 2020). These models highlight how AI frameworks can efficiently uncover complex patterns from high-dimensional transcriptomic data but at the same time could overfit. Besides this, the majority of DL models require large sample sizes, and their predictions are harder to explain.

Using the bulk and single-cell transcriptomic data allows for a wide biomarker discovery. Over 12 different biomarkers were identified in this review as predictive of immunotherapy using computational approaches. Some biomarkers are well-established and previously validated in BC, while others remain novel and require further validation. This highlights the growing interest in integrating mechanistic insights such as redox, anoikis, and palmitoylation to go beyond classical immune signatures.

However, the high number of biomarker signatures identified through AI approaches raises several concerns. Several studies relied on computational validation by statistical metrics, cross-validation, or train/validation techniques, which could be biased due to the models capturing dataset-specific patterns. Compared to these, Shen, Zhang, and Guo supported their models with immunohistochemistry (IHC) to confirm gene expression. Meanwhile, besides the IHC, Liu further added in vivo testing on a 4 T1 mouse model. With this number of biomarker signatures in the literature, researchers should compare their predictive models against existing clinically validated biomarkers to underline the value of their new signature within the clinical standards. A subset of researchers, including Shen, Ensenyat-Mendez, and Liu, validated their models/genes against PD-1/PD-L, TMB, or other known biomarkers. Notably, Guo validated the stability and accuracy of its predictive model across 83 published signatures in 9 independent BC cohorts.

The integration of transcriptomic biomarkers with AI models offers a promising path toward personalized immunotherapy in breast cancer. However, as the field evolves, several critical gaps remain to be addressed to guide more effective and clinically relevant research. TNBC is widely analyzed for its immunogenicity, while HER2-positive and luminal A/B subtypes are underrepresented. Future work should expand transcriptomic modeling to non-TNBC subtypes, including immune-low HER2 + and ER + breast cancers, where new therapies are emerging. Incorporating spatial transcriptomics and cell–cell communication analysis will offer a wider and more complete understanding of immunotherapy characteristics to refine immune subtype stratification and response prediction. Researchers should also validate their novel biological biomarkers and benchmark them against other immunotherapy biomarkers. Besides this, prioritizing public ICI-treated cohorts allows for maintaining validation consistency and enables real-world implementation, especially in the absence of experimental validation.

### AI-based proteomic analysis

Proteomic-based AI biomarkers have been explored to enhance BC prognosis and immunotherapy response prediction. PDL1 expression is an important prognostic biomarker for immunotherapy response in BC. However, many studies have demonstrated that clinical assessment by pathologists has shown a low inter-observer agreement in assessing PD-L1 expression (Reisenbichler et al. 2020; Widmaier et al. 2020). To ensure the repeatability and accuracy of the quantification of this biomarker, researchers have integrated AI technologies for PD-L1 scoring. Beyond PD-L1 prediction, several studies have explored proteomic biomarkers related to plasma for their potential to predict immunotherapy response. Table 1 summarizes the different AI/ML studies applied to proteomics in BC immunotherapy.

Deep learning models were often applied in these studies. CNN architecture was adopted by two different studies; Wang et al. used LinkNet, a CNN-based encoder-decoder architecture designed for pixel-wise segmentation, to score PD-L1 expression using IHC WSIs (Chaurasia and Culurciello 2017). The model segmented the cell, epithelium, and necrotic regions, removing strained regions overlapping with tumor and necrotic areas, and its predictions allowed for PD-L1 scoring and quantification (Wang et al. 2021). An end-to-end CNN model was adopted by Shamai et al. to predict PD-L1 expression using hematoxylin and eosin (H&E) images. The model was built with four residual blocks that encoded input images into a 256-dimensional embedding vector and a SoftMax linear layer for label classification. Its performance was validated using two methods; the prediction scores were compared to the ground truth annotation of a pathologist, and a validation based on an independent external cohort that achieved a remarkably high AUC (0.854) (Lu et al. 2020). Lastly, a model was constructed using ResNet-50-based feature extraction and integrated with an attention-based multiple instance learning (MIL) model for sample-level prediction (Ligero et al. 2024). This approach effectively captured and aggregated meaningful tile-level features, highlighting the potential of the DL-based PD-L1 scoring method to stratify patient response to immunotherapy.

In contrast, traditional ML studies were mostly used for the investigation of proteomic datasets with RF applied for plasma-related gene classification, while Lasso-Cox regression was used for the prediction of overall survival based on protein expression (Yu et al. 2023a; Li et al. 2024). While the models didn’t use complex DL methods, they were the most interpretable models with an easy clinical application.

### AI-based histopathological analysis

**Fig. 1:**
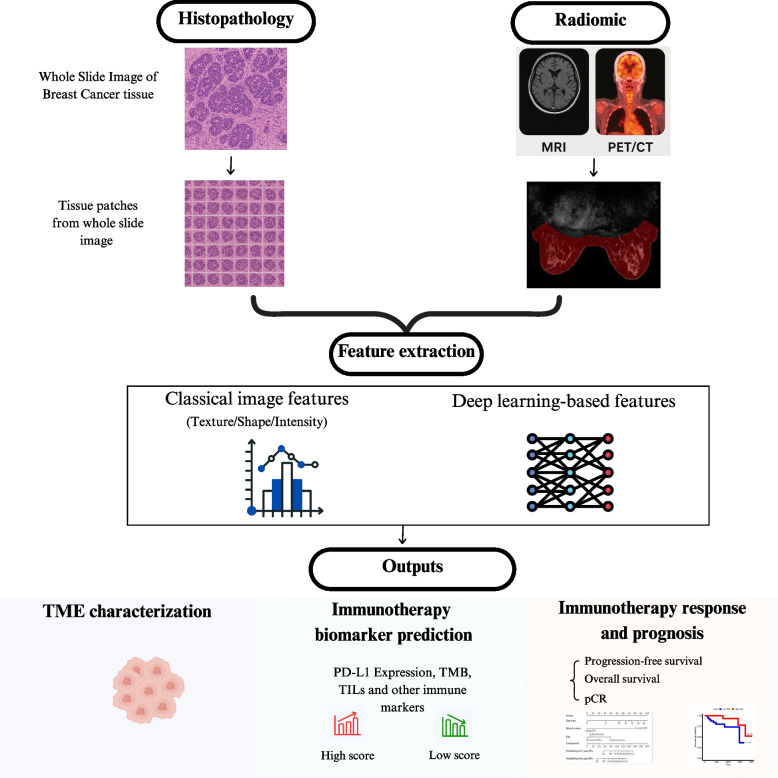
Imagery-based AI models for breast cancer immunotherapy prediction. Histopathology (WSI) and radiomic (MRI, CT/PET) images are analyzed and fed to deep learning model or other computational feature extractor to capture representative features. The model is then constructed and validated for its performance in assessing immunotherapy prognosis, identifying relevant TME characterization, and quantification of specific biomarkers. *magnetic resonance imaging (MRI), computed tomography (CT), positron emission tomography (PET), whole slide imaging (WSI), region of interest (ROI), pathological complete response (pCR), tumor-infiltrating lymphocytes (TIL), tumor microenvironment (TME), tumor mutation burden (TMB)*

H&E-stained images offer a detailed representation of the immune environment, highlighting cell morphology, tumor color, and size. These captured features make them valuable tools for AI-based prognostic models. (Figure 1) As summarized in Table 1, multiple studies have used histopathology images to extract representative features and employed AI-based models to predict immunotherapy response.

CNN models are widely used for image classification due to their convolutional backbone layers. Zhao et al. trained a dual-CNN framework for tissue classification and then predicted molecular biomarkers (somatic mutations, copy number alterations, biological pathway activities, and immunotherapy biomarkers) with AUCs ranging from 0.70 to 0.87 (Zhao et al. 2022). Another CNN-based AI model was designed to predict long non-coding RNA (lncRNA) metabolism subtypes using a bag-of-patches approach, ResNet50-based feature extraction, and an attention mechanism (Yu et al. 2024). While these DL models offer technical advantages, their clinical validation remains limited primarily due to the lack of interoperability. To address this, authors employed various bioinformatics and validation techniques to ensure the reliability and robustness of their predictions.

Despite the complexity of the CNN, we explored, in a retrospective study, the use of traditional machine learning approaches to predict immunotherapy outcomes based on TNBC WSIs. Our findings demonstrated the efficiency and reliability of RF as a non-invasive alternative to expensive TMB quantification (Bendani et al. 2025). Machine learning models, such as LR and SVM, are simple, interpretable, and highly compatible with numeric features but are often limited to the quality and relevance of the features and have limited performance. An application of tissue-based prediction used a regularized LR model trained on cell phenotype densities, cell interactions, and proliferative fractions extracted from H&E images to predict pCR. Tissue features were analyzed at distinct moments (baseline, early on treatment, and post-treatment) and reduced using Spearman correlation and Louvain clustering. The study emphasized that integrating a simple ML model with spatial features enhances immunotherapy response prediction (Wang et al. 2023a).

Due to the large size of the WSIs and the computational cost of processing them, researchers adopt substantially various approaches. Classical techniques extract the morphological and texture features (for example, nuclear shape, texture, and color gradients) using different annotation tools such as CellProfiler and Pyradiomics, and while this captures biologically interpretable features and is computationally efficient, the quality of the features and the requirement of manual curation are still a challenge. Chen et al. proposed a patch-based CNN model to predict TMB, a genomic biomarker associated with immunotherapy, in TNBC patients using nuclear scores extracted from WSIs (Chen et. al 2022). They overcome the patch-level limitation by keeping only representative patches with sufficient nuclear cell composition to achieve better performance using a pre-trained CNN architecture.

Modern approaches primarily utilized patch-level classification or MIL techniques. In patch-level classification, WSIs are divided into tiles/patches of a specific size, and then each patch is labeled, classified, and aggregated at the image level. These approaches focus on the local regions, capture small variations of images and detailed ones, and give high performance when integrated with attention mechanisms. This is highlighted in the ANSAC model study, where authors combined spatial priors, patch-level extraction, and attention mechanisms to predict TILs, demonstrating higher interpretability and scalability across different datasets (Perera et al. 2024). Moreover, adopting histology-specific pretraining improved feature quality compared to models pre-trained on natural images. Despite this, the patch-level classification models are limited due to the necessity of region-of-interest annotation and could miss global tissue patterns.

MIL is useful when patch-level labels aren't available or manual classification is costly and time-consuming. It relies on the WSI labeling and treating the images as a “bag” of instances without labels. The model is then used to learn and identify images that are important for predicting the bag label. An example of this is the BBMIL model constructed to predict classical biomarkers and immunotherapy-related gene signatures directly from histopathology images. The model utilized the ResNet-18 classifier to select a region of interest in cancer regions, generate pseudo-bags, and perform instance-level feature extraction; then it applies a transformer and attention MIL networks for the bag-level feature representation and finally predicts biomarker status. The model outperformed other MIL approaches in predicting immune signatures such as dendritic cell, interferon-gamma, and B cell signatures (Zhang et al. 2025). The MIL models, while powerful, often suffer from poor interpretability, and thus integrating attention mechanisms highlights tissue parts that drive the prediction, making results easier to validate by pathologists.

Despite this wide number of studies and promising advances, several limitations persist, with low interpretability a key challenge. Clinical validation on immunotherapy-treated cohorts and the integration of MIL, or self-supervised learning with attention mechanisms, could compensate for the limited interpretability of DL and the need for heavy data annotation while benefiting from its powerful prediction. Besides, models such as graph-based and transformer-based could offer an alternative solution for capturing both global and local tissue biomarkers (cell–cell interactions and spatial relationships). Additionally, adopting the alternative formats of images, such as tissue microarrays (TMA) and multiplex immunohistochemistry (mIHC), could provide a richer, biologically grounded, and clinically relevant prediction.

### AI-based radiomic analysis

As a non-invasive approach, radiomics has become broadly used by healthcare providers, allowing disease diagnosis and prognosis. Following this trend, it becomes a fundamental approach in precision oncology, enabling cancer identification, monitoring, and real-time treatment adaptation. Recent studies outlined in Table 1 have demonstrated the integration of AI computational models with medical imaging techniques such as magnetic resonance imaging (MRI), computed tomography (CT), and positron emission tomography (PET/CT) (Fig. 1).

The majority of the studies relied on classical ML approaches or basic DL models for the classification of the radiomic features extracted using traditional tools such as Pyradiomics or Analysis-Kit. Among the models, LR and LASSO were most used in the pCR and immunotherapy response prediction. This highlights the reliance on interpretable models that work efficiently with limited data. Huang and Lin, on the other hand, compared six ML models, including RF, SVM, MLP, and Gaussian Bayes, with leave-one-out cross-validation to predict TIL levels using 11 DCE-MRI features (Huang and Lin 2022). Interestingly, the MLP model achieved the highest predictive score, highlighting the power of the basic DL model against the ML models. Zhao et al. also achieved the highest performance with the MLP (AUC = 96%), driven by robust training data (*n* = 240) and clinical validation. They used CE-CT images to extract features and constructed the predictive model with 3 hidden layers of MLP (Zhao et al. 2023). Despite the simplicity of MLP over CNN or transformers, it's a computationally lighter model, robust in analyzing non-linear and numerical data as radiomic features. The most innovative approach was used by Cook et al. in their study incorporating biophysics-based computational modeling. They introduced the tumorIO prognostic score to predict immunotherapy response based on DCE-MRI and single-cell RNA sequencing data. A simul-omics 4D engine segmented MRI scans with a CNN, simulated blood flow, nutrient delivery, and metabolic activity, and integrated them with the transcriptomic features related to PD-L1 expression to produce spatial probability maps of PD-L1 expression. Then, a linear regression model generated the score to predict pCR following ICI therapy (Cook et al. 2023). These approaches offered several advantages, including biological interpretability and spatial modeling, which achieved high performance (ACC = 88.2%) despite the limited size of the dataset.

Radiomics data presented in the reviewed studies encompass the features extracted from CT, MRI, and PET-CT, with MRI being the common imaging modality used. Models constructed using the MRI features achieved a mean AUC higher than 80%, highlighting the ability of the MRI to capture biological features associated with immunotherapy response, such as perfusion, vascularity, and microenvironmental heterogeneity. Other than MRI, contrast-enhanced CT was used by Zhao et al. to extract texture, wavelet, and Laplacian of Gaussian features, while Seban et al. combined CT with PET, which measured metabolic features (SUVmax, TMTV) rather than classical radiomic patterns (Zhao et al. 2023; Seban et al. 2023) As these imaging techniques offer a high dimensionality of radiomic features, feature selection is crucial to accurately extract representative ones and avoid feeding noise or overfitting the predictive models. Statistical techniques such as LASSO, Boruta, and univariate correlation analysis are common feature selection techniques and well-suited for selecting clinically significant features due to their interpretability, computational efficiency, and regularization techniques, while deep learning models such as autoencoders and MLPs can capture complex, non-linear patterns associated with class prediction, although at the cost of reduced interpretability. Biological filtering was also applied by other studies to extract only features associated with biological signals such as TME or PD-L1.

In summary, most radiomic studies on breast cancer immunotherapy have leaned toward traditional methods with relatively simple ML models. Deep learning has mostly been limited to MLPs, CNNs and attention-based architectures mainly due to the dataset sizes and the focus on more interpretable models. However, future studies should integrate DL architectures in feature extraction and selection and balance between interpretability and model complexity to construct models capable of the analysis of the heterogeneous immune system in BC. Researchers should also use other imaging modalities, such as ultrasound, which offer real-time monitoring and are low-cost and accessible. Additionally, integrating raw images with basic radiomic features can enhance the model's performance, offering a way to learn high levels of abstractions while keeping the interpretability.

### AI-based microbiomic analysis

The microbiome influences cancer diagnosis, treatment, and progression (Tzeng et al. 2021; Vitorino et al. 2022; Clear et al. 2024). Despite the increasing interest in the tumor microbiome, we identified only two studies, summarized in Table 1, employing AI-based models to identify microbiome signatures related to BC immunotherapy and predict patient outcomes.

Mao et al. identified a set of 15 microbes using Cox regression and constructed a prognostic marker based on their abundance. These signatures were identified after univariate Cox regression as a first step to reduce dimension, followed by a second reduction using multivariate Cox regression. This two-step reduction is well established in survival analysis, especially with highly dimensional data. Univariate Cox is faster in screening individual features associated with survival, while multivariate Cox controls for confounding and identifies the most robust predictors. The predictive score generated was significantly associated with overall survival (OS) and progression-free survival (PFS) with a p-value of 1.70E-19 and 5.27E-06, respectively. They also created a nomogram to compute a risk score by integrating the microbe signatures, clinical factors, and molecular subtypes with an AUC of 0.80 in the 5-year OS prediction (Mao et al. 2022).

Li et al. adopted a similar approach for selecting immune-related intratumor microbiome biomarkers for BC patients’ OS prediction. He used LASSO and multivariate Cox regression to build a prognostic signature score consisting of the expression of four genera, such as *acidibacillus*, and *pseudogulbenkiania*, which were associated with antigen processing and anti-tumor immunity by regulating the immune-related genes. Based on the immunophenotype scores and tumor immune dysfunction and exclusion analysis, patients with low-risk scores could benefit more from immunotherapy than the high-risk patients (Li et al. 2024).

Both studies focused on first-level microbiome features such as microbial abundance and relied entirely on the TCGA dataset. Statistical ML techniques, mainly Cox regression, were solely used without integrating DL or complex approaches. While one study demonstrated the biological relevance of these signatures using bioinformatic analysis, none of them validated their findings experimentally. Cross-disciplinary efforts are needed to unlock the full potential of microbiome data in the prediction of immunotherapy. Future research should expand feature diversity and integrate deep learning models to uncover complex microbe-immune associations without neglecting the preprocessing and contamination control to allow for reproducibility and clinical application.

### AI-based genomic analysis

Genomic biomarkers include copy number alterations (CNA), neoantigen load, and genetic variation. These biomarkers can be used to describe immunogenicity and thus predict response to immunotherapy. Various studies have been investigating the integration of AI models with genomic biomarkers, as presented in Table 1.

Across these studies, distinct ML and DL models were created, ranging from interpretable classical ML like Cox regression to complex neural networks. A pan-cancer study trained different machine learning classifiers to predict immunogenic mutations and neo-peptides. Among the models tested, LR performed the best, especially with increasing data, adding quantile normalization, and Bayesian optimization. XGBoost, while ranking second, identified different and complex patterns, and when combined with LR in a voting classifier, achieved consistently higher performance (Müller et al. 2023). Jin et al. created a prognostic risk model to predict genomic variation-related subtypes by feeding DEG genes to LASSO-penalized multivariate regression with 1,000 iterations (Jin et al. 2021). Similarly, Jiao et al. performed multivariate Cox regression on lncRNA genes to stratify patients as genomically unstable or stable (Jiao et al. 2022). Both models achieved similar performance, with the LASSO-personalized multivariate model slightly higher. This is primarily due to the regularization technique and penalty used by LASSO during model training, which automatically deals with multicollinearity while reducing overfitting and selecting important features.

The RCANE model, combining sequence models and graph neural networks, was developed in a pan-cancer cohort to predict somatic CNA from RNAseq. 3D tensors, in which somatic CNA log-intensity values are synthesized per segment, were used to define the segment-based graph capturing inter-chromosomal, and MLP with normalization is then used to extract cancer-specific RNA features. LSTM and graph attention mechanisms are integrated into the model to detect cross-chromosomal correlation, and a debiasing layer with a regression loss function predicts sCNA intensity. This DL model outperformed existing methods, achieving high accuracy and highlighting the potential of the DL model as a cost-effective alternative to traditional sequencing-based methods for SCNA detection (Ge et. al 2024). To advance the field, future studies should further analyze genomic biomarkers and investigate the combination of DL models with the interpretability of statistical techniques.

### AI-based epigenetic analysis

BC prognosis is associated with epigenetic modifications, such as DNA methylation, histone modifications, and non-coding RNA expression. These alterations can impact diverse immune-related pathways, regulating tumor immunogenicity and affecting immunotherapy effectiveness (Llinàs-Arias et al. 2021; Yin et al. 2023). Machine learning, statistical methods, and deep learning have been employed to extract and predict immunotherapy by leveraging epigenetic biomarkers. (Table 1) For instance, DNA methylation, particularly at CpG sites, is a well-established epigenetic factor that influences gene expression and immune response in cancer (Zhu et al. 2024b).

Various models, validation strategies, and biomarkers were used to identify prognostic and immunotherapy-relevant biomarkers in BC. Zhang created a CpG methylation risk score to stratify patients into high- and low-risk groups. Despite the CpG methylation being associated with immune natural killer cell infiltrate, particularly at specific CpG sites, LASSO achieved a moderate accuracy, highlighting the need for a more complex model and feature selection methods (Triki et al. 2022). Chen, on the other hand, focused on the immune cell-specific hypermethylation signatures, achieving high performances (AUC = 0.86) with LASSO. This demonstrates that data preprocessing and feature selection tend to optimize model prediction. Beyond nuclear epigenetics, mitochondrial epigenetics has emerged as a regulator of the tumor. A model study investigated mitochondrial DNA methylation with immunotherapy response in BC and proposed a gene signature to predict BC prognosis. They identified 11 mtDNA methylation-related genes and established a signature score after applying univariate Cox analysis and LASSO. Notably, they used qPCR to validate their findings and verified the TMB, CD8, and MSI (Ma et al. 2023). Besides this, Teng focused on RNA methylation and constructed a 21-methylation regulatory gene signature to stratify BC patients (Teng et al. 2024). This emerging field is promising but needs further clinical validation.

lncRNAs, a novel class of ncRNAs, have been involved in the immune response of BC (Zhang et al. 2020). Researchers have explored several lncRNA-based prognostic signatures to construct a risk model using multivariate, univariate Cox regression, and LASSO (Zhou et al. 2024; Zhu et al. 2022; Li et al. 2022; Yu et al. 2023b; Chen et al. 2022b). Among these studies, Yu exploited the senescence-related lncRNAs that were linked to immunotherapy resistance by upregulating immunoinhibitory proteins, including PD-L1 and CD80. Its model achieved the highest AUC (81.1%) compared to the rest of the LASSO-based models (Yu et al. 2023b). This suggests that while being the most interpretable and clinically used models, they achieved moderate performance. An interesting study diverged from traditional LASSO approaches by testing six ML algorithms (DT, GBM, ANN, RF, GLM, and SVM) to predict CD8 + T-cell levels using lncRNAs (Chen et al. 2022b). The models achieved a range of AUCs, with ANN the highest among them, highlighting the potential of deep learning models in capturing non-linear interactions between lncRNAs and immune infiltration.

Trends across these studies suggest a shift from whole methylation profiling to a more specific approach with cell-specific methylation analysis as mitochondrial and immune cell-specific biomarkers. Future studies should also emphasize the clinical validation of their signature and apply them to the immunotherapy cohort.

### AI-based multi-omic analysis

**Fig. 2:**
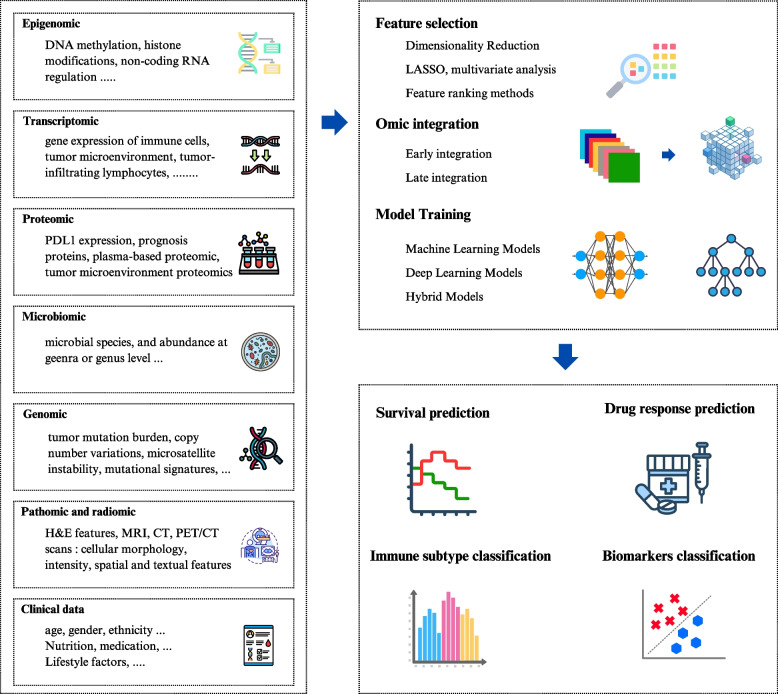
Multi-omic integration in breast cancer immunotherapy. The multi-omic approach combines various biomarkers from epigenomic, transcriptomic, proteomic, microbiomic, genomic, radiomic, and clinical data. After feature selection and data integration, the model is constructed to predict survival, drug response, immune subtypes, and biomarker classifications in breast cancer immunotherapy. Least absolute shrinkage and selection operator (LASSO), magnetic resonance imaging (MRI), computed tomography (CT), positron emission tomography (PET), hematoxylin and eosin (H&E)

Multi-omics integrates diverse data, particularly genomics, epigenetics, radiomics, and transcriptomics, providing a comprehensive understanding of cancer biology. Given the heterogeneous nature of BC, single-modality models cannot capture the full complexity of the disease. In contrast, multimodal architectures overcome these limitations by combining multiple data layers, allowing for more precise and personalized prognostic assessments. Several methods were developed to accurately capture the complex patterns and contribute to the evaluation and prediction of immunotherapy. Some of these models are presented in Table 1 with a detailed workflow summarized in Fig. 2.

ML and statistical models are widely used for their strong interpretability, efficiency, and integration of structured and numerical omics. A notable instance is the iCEMIGE model, which independently analyzed cell morphometrics, bacterial diversity data, and transcriptomic biomarkers to generate an integrated risk score. The morphological cell features were extracted using an unsupervised sparse decomposition pipeline and were combined with microbiome and gene prognostic scores previously identified using multivariate Cox regression. The integration was performed with an autoencoder and sparse representation learning into 256 multimodal biomarkers, ensuring interpretability and efficiency in the selection of biomarkers. Using multivariate Cox regression, a risk score was constructed and achieved higher predictive performance in predicting both OS and PFS compared to each modality alone (Mao et al. 2022). Similarly, a radiogenomic prognostic score was constructed to predict axillary lymph node metastasis and drug response, integrating MRI-derived radiomic features and genomic data. The prognostic model was based on a linear SVM with an RFE algorithm feature selection, followed by multivariate LR analysis to generate the risk score, which, when high, indicates a potentially better response to targetable immunotherapy (Lai et al. 2024). A recent study by Mehmood et al. investigated single- and multi-omics models to predict survival class based on transcriptomic, proteomic, and clinical data. Supervised ML models such as SVM, Naive Bayes (NB), and LR with MLP DL model were applied for single omic prediction, with MLP achieving higher performance. Regarding the combined multi-omic data, a multi-source feature interaction controller was adopted to handle the different omic types with a unique model for each omic and a fusion model to integrate them for final prediction (Mehmood et al. 2025).

DL models, on the other hand, dominate when the input data are complex, highly dimensional, or time-related. DeepClinMed-PGM was developed by integrating several features, among them lncRNA data, pathomic features, immune cell levels, and personal features. The model predicted survival risk using a CNN architecture integrating an attention module, a regressor, and a query-router mechanism to enhance the accuracy of patient prognosis. Notably, its superior performance over the single-omics model was consistent across different validation cohorts. By assigning distinct weights to the different modalities, the model captures complex relationships, reinforcing its capability to provide more precise prognostic predictions (Yu et al. 2024). Furthermore, a deep learning-based model was developed by Gao et al. to predict therapy response in BC by integrating omics data, including imaging, alongside radiological, histopathological, personal, and clinical data. This model addresses the problem of missing data by leveraging cross-modal knowledge mining, enabling clinical, personal, and other data to be predicted directly from imaging-derived features. A ResNet18 architecture was used to extract features with temporal information added for the MRI model, and their predictions were combined using an ensemble model using optimized weightings to improve the overall prediction accuracy. As expected, the model's predictive capacity outperformed radiologists in early therapy response prediction (Gao et al. 2024).

Expanding beyond BC, a pan-cancer study investigated the gene mitotic apparatus signature, linking it to immune cell infiltration and treatment response in glioma and BC. Researchers further identified cellular morphometric biomarkers extracted from WSIs using a stacked predictive sparse decomposition model and constructed a signature score using multivariate Cox Proportional Hazards. The integrated model significantly improved prognostic precision, demonstrating its potential for patient risk stratification (Mao et al. 2023).

### Computational modeling in breast cancer immunotherapy

Computational modeling (CM) has revolutionized breast cancer therapy advancement, especially in immunotherapy. This approach allows the modeling of drug-drug interactions, the discovery of drugs, immune pathway simulations, and virtual drug screening, providing insights into the efficiency of the immunotherapy compound. When integrated with AI, these models can predict drug response, simulate immune invasion, and predict novel biomarkers. This integration enables researchers to explore new compound interactions, refine therapies, and predict responses to these therapeutic strategies.

Molecular docking, a known cornerstone of computational modeling, predicts the interaction between a drug or ligand with a target (receptor or enzyme). Applied in immunotherapy, this provides insights into how molecules such as PD-1/PD-L1 or CTLA-4 could be targeted to enhance immunogenicity. Traditional studies use a combination of computationally heavy and time-intensive approaches to molecular docking simulation and drug repositioning. As an example, a study used computational drug repositioning, reversal of gene expression signatures, and molecular dynamics simulations to identify and validate potential drug candidates for BC treatment (Wang et al. 2023b). While this approach successfully identified new compounds as potential BC treatments, applying AI can further improve and optimize the study. From using DL models to model drug and gene networks and autoencoders for data reduction to DNN architecture for molecular interactions, AI can be integrated into the entire computational modeling workflow. A recent study applied supervised computational screening of EGFR inhibitors to identify molecules capable of inhibiting immune checkpoints. BC omics, including scRNA-seq and genomic data, were computed for drug sensitivity and IC50 analysis to select a strong EGFR inhibitor. Next, compounds similar to the selected reference drug were fed to different ML models to predict and screen active candidate compounds against EGFR. RF, SVM, and ANN achieved high performance (AUC up to 94%), highlighting AI potential in enabling fast identification of inhibitors and supporting drug repurposing. These findings were further validated by molecular docking, providing a roadmap for designing immune-activating drugs (Mehmood et al. 2024). Another study employed ML-based approaches to predict the anticancer activity (IC50 values) of terpenes and their derivatives against specific TNBC cells. A robust QSAR-based multiple linear regression was constructed using forward stepwise regression to select the best combination of 2D molecular descriptors with minimum multicollinearity with a predictive r^2^ of 81%. The resulting model was implemented as an R-based package to allow researchers to predict IC50 values of novel, untested chemicals and understand descriptors associated with anti-TNBC activity (Khan et al. 2024). In addition to screening molecules and predicting activity, AI can predict binding affinities and identify novel ligands based on their pharmacodynamics.

Combination therapy has improved outcomes, responses, and resistance in BC by targeting diverse pathways simultaneously. In a recent study, a stacking ensemble classifier called DDSBC predicted synergistic drug pairs in breast cancer cell lines based on transcriptomic and chemical data. The authors aggregated predictions from base models (RF, XGBoost, KNN, and LR) for a robust prediction. The model was trained on a vast dataset of drug responses and achieved good performance in predicting drug-pair combinations. Such AI-based tools enable rapid and accurate screening of thousands of drug combinations for optimal immune enhancement with minimal toxicity (Mehmood et al. 2024).

Pharmacogenomics aims to tailor specific drug therapy based on a patient's genetic variation. This field, a core part of personalized medicine, has drastically improved the selection of effective immunotherapy strategies, especially with the aid of AI. A notable example of AI-powered models is a study that applied machine learning models (LASSO, Elastic Net, and Ridge) with transcriptomic and molecular profiling data to predict drug sensitivity across breast cell lines. Feature selection was performed using maximum relevance and minimum redundancy to reduce dimensionality and improve interpretability. The authors successfully predicted candidate drugs and identified new translationally relevant biomarkers (Mehmood et al. 2023).

### Nanotechnology in breast cancer immunotherapy

Combining nanotechnology with immunotherapy agents enhances the response by enabling the manipulation of materials of small size (< 100 nm). Adopting nanoparticles (NPs) improves a targeted distribution of drugs while protecting other healthy tissue by offering biocompatibility, the possibility to load a high number of drugs, and a longer circulation time. Currently, several nanomaterials, such as liposomes, microneedles, polymer-based conjugates, and dendrimers, are applied to improve cancer immune response. Multifunctional nanoplatforms like P-R@P/U-V and PD-1@RSL3 NPs were developed by combining immunotherapy mechanisms, BC biomarkers, and nanotechnology. For instance, P-R@P/U-V combines tumor ablation, ferroptosis, and real-time imaging to enhance anti-PD-L1 immunotherapy in TNBC, representing a next-generation nanomedicine approach. The laser-activated combat TNBC cells, while ferroptosis induction enhances the release of tumor-associated antigens. The combination of this local tumor destruction with anti-PD-L1 therapy boosts antigen release and immune activation and suppresses metastasis (Cheng et al. 2024). Another example is the PD-1@RSL3 NPs, PD-1 membrane-coated RSL3 nanoparticles developed to enhance cancer immunotherapy, especially in TNBC. These nanocarriers bind to the PD-L1 expressed on tumor cells and block immune checkpoints, reactivating T cells, and at the same time, the RSL3 triggers ferroptosis cell death, leading to the release of tumor antigens and enhancing the immunogenicity. In animal subjects, this combination of PD-L1 blockade and ferroptosis induction leads to a robust immune response, with a significant delay in tumor progression and extended survival (Mu et al. 2023). A dendritic-inspired nanoparticle, DMSNs^3^@HA, combines anti-CD3 and anti-CD28 to activate T cells, anti-PD-1 antibodies for checkpoint blockade, and hyaluronic acid for tumor targeting (Li et al. 2021).

Nanotechnology was applied to monitor immunotherapy response. The electrochemical biosensor's role is to convert biological interactions into electrical signals, allowing real-time, minimally invasive detection of specific biomarkers. Combining nanomaterials such as carbon nanotubes with electrochemical biosensors enhances biocompatibility and signal amplification, making them powerful tools for monitoring immunotherapy response (Sadeghi et. al 2023). By detecting sensitive changes in biomarker expression, clinicians could monitor, adjust, and evaluate patients' responses to immunotherapy treatments. A noticeable trend is the emergence of nanoplatforms combining multimodal therapy with nanomaterial technologies. For example, DoxMel/PD-L1 DsiRNA proposed by Bahreyni et al., integrates chemotherapy (doxorubicin), immunotherapy (PD-L1 RNAi checkpoint blockade), and an immune adjuvant in hyaluronic acid for targeting tumors via CD44. By adopting this efficient delivery of 3 different modalities, survival was improved with a significant enhancement of cytotoxicity and cell infiltration (Bahreyni et al. 2023). Similarly, a study developed ICG@SANPs-cRGD, a multifunctional iron-based nanoplatform combining photothermal therapy (PTT), photodynamic therapy (PDT), and immune checkpoint blockade (anti-PD-L1). These nanoplatforms not only amplify anti-tumor immunity but also demonstrate imaging capabilities (MRI and fluorescence), enabling theranostic applications (Kong et al. 2022). Another application is the nanovaccines used to stimulate T-cells, reshape the tumor immune microenvironment, and enhance the durability of the immunotherapy effect. They can target lymph nodes, tumor antigens, mRNA, and biomimetics, allowing for an adaptable and precise application (Yin et al. 2020).

AI models can advance the design of nano-drug delivery systems by analyzing the immune cell and cytotoxicity profiles based on the physicochemical properties of the nanoparticles. Furthermore, AI can be used to create personalized nanovaccines by analyzing patient omics data and predicting specific neoantigens, thus offering a personalized and optimal response. AI can also evaluate signals from various biomarkers, such as immunotherapy biomarkers, cancer progression biomarkers, and metabolic changes, in real time by integrating electrochemical biosensors. This allows for the classification and prediction of changes in these biomaterials, allowing for the detection of resistance or the prediction of early response. Generative AI and reinforcement learning could be used to explore new nanomaterial structures and suggest novel surface modifications. Additionally, imaging-based nanoparticles offer a real-time and efficient modification of delivery routes due to tissue damage or patient complications.

### The AI-driven healthcare landscape

Recently, the integration of automated learning systems and precision medicine has transformed healthcare, particularly in the field of oncology. Personalized medicine relies on the usage of tailored treatment for cancer patients by adopting a more individualized approach that considers the patient's distinctive characteristics. This has transformed the conventional “one treatment fits all” paradigm, allowing a more individualized medicine (Johnson et al. 2021). Given that BC is distinguished by a significant heterogeneity among patients, healthcare providers should take into account the patient’s genetic profile, medical background, environmental factors, and lifestyle preferences. AI offers a powerful potential to enhance the analysis of data, uncover complex patterns, and refine treatment strategies. Various AI models have been developed, as described in this review, to predict therapy outcomes through multimodal methods that integrate diverse patient data from clinical, genomic, epigenetic, proteomic, and microbiome information. AI-powered technologies, such as wearable electronics and biosensors, reshaped cancer care by providing continuous monitoring of patient parameters and offering non-invasive and accurate methods for diagnosis (Birla et. al 2025). An example of these devices are smartwatches and smart patches, which, simple as they are, allow heart rate, respiratory, and glucose tracking, offering non-invasive monitoring. Nanosensors, on the other hand, can detect biomarkers at the molecular level, such as ctDNA or proteins, and are usually integrated into wearable devices to enable hand-free and continuous quantification of biomarkers (Adeniyi et al. 2024).

With such advancements in AI-based wearable nanodevices capable of monitoring, analyzing, and adjusting treatment, the role of healthcare providers must be redefined. This prompts a reconsideration of how health professionals contribute to patient care within an AI-enhanced healthcare ecosystem. While AI-based systems can process heterogeneous data and identify complex patterns in a reasonable time, they lack the human ability to consider the patient's quality of life and personal and economic considerations. Furthermore, AI predictions may be skewed for several reasons, including differences in demographics that lead to wrong choices and predictions. To address these challenges, an interdisciplinary team must validate AI-driven predictions to ensure reliability and collaboratively establish a treatment plan with the patient, taking into account their preferences and values. This approach is consistent with the concept of shared decision-making, utilizing AI as a support tool that analyzes data and provides recommendations to help guide discussions between patients and healthcare providers (Seyhan and Carini 2019).

**Fig. 3:**
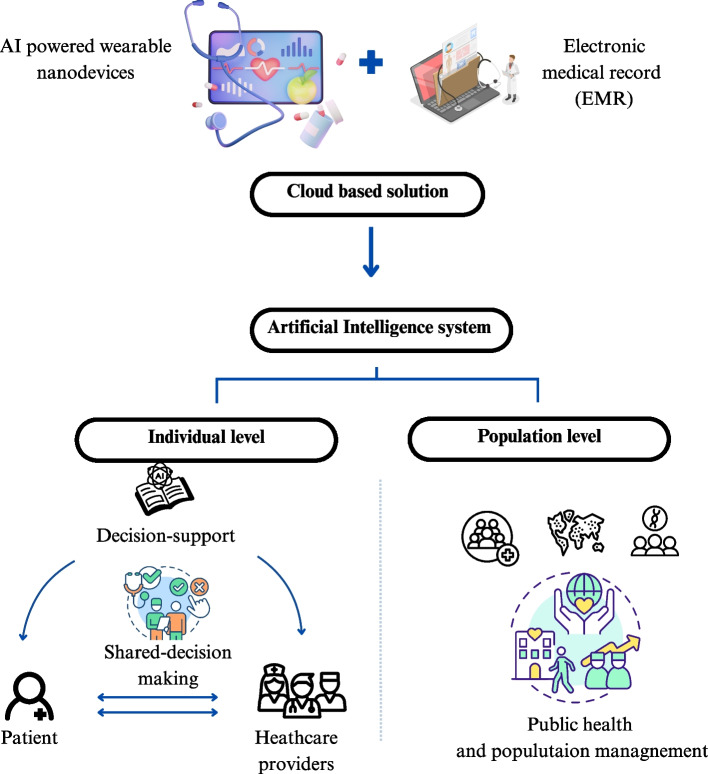
Integrated AI-driven healthcare ecosystem from personalized medicine to public health management. AI-powered wearable nanodevices and patient medical records are integrated in complex databases and stored in a cloud-based solution. The AI system analyzes this data as omic to generate personalized treatment recommendations tailored to the patient. These recommendations are then integrated through the shared decision-making system by providing it to both the patient and the healthcare provider. Beyond individual care, this system produces valuable big data that supports public health initiatives by enabling disease surveillance, and optimized population health management

The integration of personalized medicine, AI nanodevices, and omics data with cloud-based solutions will enhance disease treatment efficacy and reinforce public health initiatives. As shown in Fig. 3, AI-based wearable nanodevices that collect real-time omics data and patient medical records are integrated into a complex system and stored in a secure cloud-based solution. These diverse data are then analyzed by AI systems, which generate personalized treatment recommendations for both patients and healthcare providers. Through a collaborative approach, patients and healthcare professionals are both engaged in shared decision-making, balancing AI insights with clinical judgment and patient preferences. A personalized and automated treatment administration can be adopted through nanodevices without the need for human intervention. Additionally, with the generation of big data that AI models can further analyze, the optimization of population health management strategies through resource allocation can be performed.

### Limitations and future directions

Patient-level immunotherapy decisions using AI models by integrating multi-omic data for better tumor profiling are the primary application of AI in personalized immunotherapy. AI also advanced the discovery of new immunotherapy biomarkers and the in vitro simulation of immunotherapy adverse effects. Although various studies have investigated the application of AI in predicting response to immunotherapy, challenges associated with their application in clinical practice remain.

One of the primary limitations is the insufficient amount of available data, as few curated datasets are publicly available. The construction of robust and accurate AI models depends primarily on the data quality, size, annotation, and preprocessing techniques applied. Training a model on biased or incomplete datasets may affect the generalization of the model, causing false predictions across diverse ethnicities and cancer subtypes comprising clinical reliability. Researchers have applied several techniques to compute missing data and balance the datasets, such as statistical imputation methods inspired by KNN, multivariate and distribution metrics, or machine learning-based imputations such as PCA, RF, or XGBoost. Using generative adversarial networks (GANs) or learnable embeddings in deep learning models offers an alternative solution for obtaining accurate results. However, these approaches are computationally intensive and may be biased by the specific training data.

Lack of standardization of data preprocessing, storage, and annotation of data affects model performance. In BC, several imaging techniques, sequencing technologies, and the non-uniform format of electronic health records make AI models highly dependent on the institution where they were developed. Moreover, privacy regulations (such as HIPAA and GDPR) often restrict data sharing, limiting the size and diversity of the datasets required for robust model training. This institutional collaboration is needed to overcome privacy issues without compromising data security, ensuring the collection of data from underrepresented populations to ensure equity. Additionally, most AI models operate as “black boxes,” with no interpretability or a clear explanation of the decision-making process. This undermines clinical transparency and further complicates the integration of AI in clinical oncology. Efforts should be made to find explainable AI, such as attention maps or SHAP, to aid physicians in understanding why an AI system recommends a specific immunotherapy strategy or predicts a specific outcome.

Limited clinical validation and integration of the AI approach in real-world cases is yet another challenge spanning both technical and ethical dimensions. Rigorous validation, multicenter trials, and collaboration between clinicians and AI developers are needed to ensure model efficiency and facilitate integration in hospital systems. Interdisciplinary collaboration between developers, clinicians, ethicists, regulators, and patients will be critical to building AI systems that are not only powerful but also inclusive.

The use of nanomedicine for the prediction and characterization of breast cancer immunotherapy has made significant advancements in recent years. Integrating digital, biological, chemical, and physical elements into a small and compact technology, has made it possible to develop various solutions. Although there are few clinical trials, researchers have validated these nanotechnologies in vitro or on animals to confirm their effectiveness. As nanoparticles vary drastically based on AI integration, their biological and physical properties, adaptive clinical trials are indispensable for regulating these solutions. Efforts are needed to create rapid and advanced minimum standards for approval, ensuring safe clinical translation.

Besides this, several challenges still need to be resolved. For instance, the heterogeneity of BC makes nanotherapy solutions less effective, especially over the resistance mechanisms. Nanoparticles must be personalized and combined with powerful predictive biomarkers to account for tumor evolution. Biocompatibility remains the central issue in nanotechnology-based immunotherapy. Nanoparticles use various materials from metals, polymers, or carbon-based materials that can induce an immune response , off-target toxicity, or even the accumulation of toxins in organs due to poor clearance. Metallic nanoparticles, for example, can interfere with specific cellular redox status, leading to oxidative stress or other complications. Propulsion mechanisms used by some nanorobots can often be toxic at the physiological level, and thus alternative energy sources still need investigation and thorough validation for their safety and biodegradability. The non-biodegradable nanoparticles can also activate innate immunity, triggering an autoimmune response or immunosuppression and reducing the efficacy of the treatment.

Nanotechnology and AI integration will help overcome some nanotechnology challenges but at other costs. Leveraging AI to tailor and design nanovaccines based on each patient’s genetic, transcriptomic, and immunologic profiles will increase the precision and therapeutic response, reducing toxicity. Furthermore, integrating electrochemical and optical nanobiosensors with AI allows for rapid decision-making and therapy adjustment. Smart nanocarriers equipped with AI can address clinical safety and regulatory approval concerns by predicting toxicity and immunogenicity. The future of AI-enabled nanomedicine requires addressing other concerns such as a lack of explainability of AI decisions and data privacy. Resolving these limitations requires multidisciplinary approaches to tackle computational modeling, clinical practice, physics, and immunology. Future research should focus on the investigation of biomimetic strategies, biodegradable materials, and non-toxic propulsion methods to overcome nanotechnology challenges and ensure safe and effective translation into clinical practice. Early and rigorous testing, integration of predictive AI-based toxicity models, and interdisciplinary design frameworks are key strategies to provide clearer guidance for future studies in this field and also to improve the safety, efficacy, and clinical translation of nano-AI approaches in cancer immunotherapy.

## Conclusions

In this review, we explored the application of AI-driven models with genomic, microbiomic, epigenetic, transcriptomic, clinical, and imagery biomarkers in predicting immunotherapy outcomes. Various models were developed to analyze single modalities and assess immunotherapy responses with moderate accuracy in several cohorts. However, we demonstrated the efficiency of combining these biomarkers in multimodal AI models to capture the complex immunotherapy-related biological system. In the era of personalized medicine, future research should focus on leveraging multimodal algorithms to optimize individualized immunotherapy and improve patient quality of life and prognosis.

Providing AI models with access to a continuous range of diverse data sources allows the conception of AI-based decision-making systems capable of monitoring treatment responses, predicting prognosis, and providing personalized treatments. This is achieved through cloud-based wearable nanodevices, capable of storing, analyzing data, and providing real-time feedback and recommendations. A thorough understanding of AI systems and their prediction algorithms is crucial for ensuring adequate follow-up by healthcare providers. The long-term prospects of this integration of AI in the healthcare system are very promising, especially with the technological advances and the increasing availability multi-omics data, introducing a new era for enhancing personalized medicine and ultimately public health management.

## Data Availability

No datasets were generated or analysed during the current study.

## Abbreviations

BC: Breast cancer
ER: Estrogen receptor
Ki-67: Antigen Ki-67
HER2: Human epidermal growth factor receptor 2
PR: Progesterone receptor
TNBC: Triple-negative breast cancer
ICIs: Immune checkpoint inhibitors
PD-1: Programmed cell death receptor 1
PD-L1: Programmed cell death 1 ligand 1
CTLA-4: Cytotoxic T lymphocyte-associated antigen
LAG-3: Lymphocyte activation gene-3
irAEs: Immune-related adverse effects
TILs: Tumor-infiltrating lymphocytes
TMB: Tumor mutation burden
MSI: Microsatellite instability
AI: Artificial intelligence
DEGs: Differentially expressed genes
RF: Random forest
MLP: Multilayer perceptron
SVM: Support vector machine
AUC: Area under the curve
TME: Tumor microenvironment
TIME: Tumor immune microenvironment
RSF: Random survival forest
ML: Machine learning
pCR: Pathological complete response
RD: Residual disease
ANN: Artificial neural networks
LR: Logistic regression
H&E: Hematoxylin and eosin
CNN: Convolutional neural network
MIL: Multiple instance learning
IHC: Immunohistochemistry
WSI: Whole slide imaging
lncRNA: Long non-coding RNA
MRI: Magnetic resonance imaging
CT: Computed tomography
PET: Positron emission tomography
TMTV: Total metabolic tumor volume
OS: Overall survival
PFS: Progression-free survival
CNA: Copy number alterations
